# [^99m^Tc]Technetium-Labeled Niosomes: Radiolabeling, Quality Control, and *In Vitro* Evaluation

**DOI:** 10.1021/acsomega.2c06179

**Published:** 2023-02-08

**Authors:** Meliha Ekinci, Emine Esin Çalışkan, Burak Çakar, Derya İlem-Özdemir, Yiğit Uyanıkgil, Emel Öykü Çetin Uyanıkgil

**Affiliations:** † Faculty of Pharmacy, Department of Radiopharmacy, Ege University, Bornova, 35040 Izmir, Türkiye; ‡ Faculty of Pharmacy, Department of Pharmaceutical Technology, Department of Biopharmaceutics and Pharmacokinetics, Ege University, 35100 Izmir, Türkiye; § Faculty of Medicine, Department of Histology and Embryology, Ege University, 35040 Izmir, Türkiye; ∥ Health Science Institute, Department of Stem Cell, Ege University, 35040 Izmir, Türkiye; ⊥ Cord Blood, Cell and Tissue Research and Application Centre, Ege University, 35040 Izmir, Türkiye

## Abstract

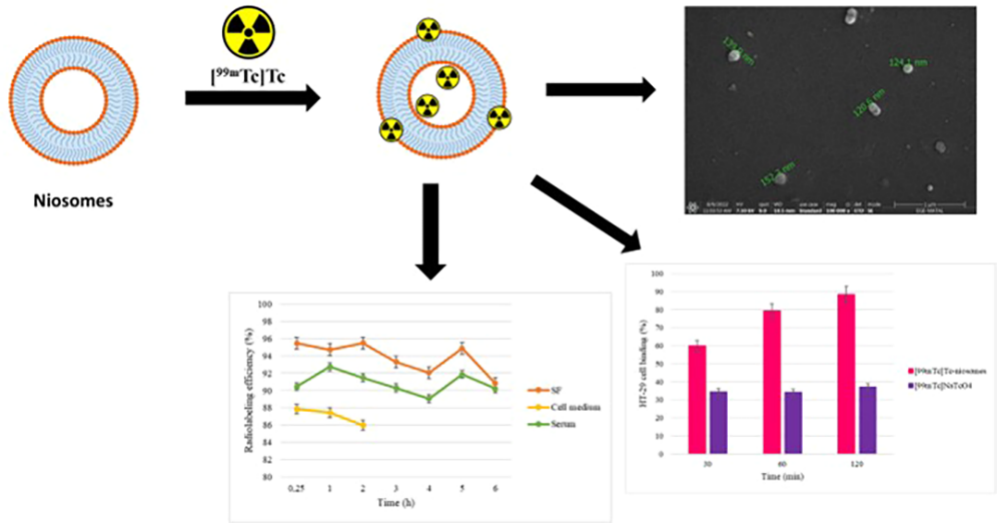

The aim of this research was to develop technetium-99m ([^99m^Tc]Tc)-radiolabeled niosomes and evaluate the cancer cell incorporation capacity of radiolabeled niosomes. For this purpose, niosome formulations were developed by film hydration method, and prepared niosomes were characterized to particle size, polydispersity index (PdI), ζ-potential value, and image profile. Then, niosomes were radiolabeled with [^99m^Tc]Tc using stannous salts (chloride) as a reducing agent. The radiochemical purity (RP) and stability in different mediums of the niosomes were assessed by ascending radioactive thin-layer chromatography (RTLC) and radioactive ultrahigh-performance liquid chromatography (R-UPLC) methods. Also, the partition coefficient value of radiolabeled niosomes was determined. The cell incorporation of [^99m^Tc]Tc-labeled niosome formulations, as well as reduced/hydrolyzed (R/H)-[^99m^Tc]NaTcO_4_ in the HT-29 (human colorectal adenocarcinoma) cells, was then assessed. According to the obtained results, the spherical niosomes had a particle size of 130.5 ± 1.364 nm, a PdI value of 0.250 ± 0.023, and a negative charge of −35.4 ± 1.06 mV. The niosome formulations were effectively radiolabeled with [^99m^Tc]Tc using 500 μg mL^–1^ stannous chloride for 15 min, and RP was found to be over 95%. [^99m^Tc]Tc-niosomes showed good *in vitro* stability in every system for up to 6 h. The log *P* value of radiolabeled niosomes was found as −0.66 ± 0.02. Compared to R/H-[^99m^Tc]NaTcO_4_ (34.18 ± 1.56%), the incorporation percentages of [^99m^Tc]Tc-niosomes (88.45 ± 2.54%) were shown to be higher in cancer cells. In conclusion, the newly developed [^99m^Tc]Tc-niosomes showed good prototype for potential use in nuclear medicine imaging in the near future. However, further investigations, such as drug encapsulation and biodistribution studies, should be performed, and our studies are continuing.

## 1 Introduction

Nanomedicine has become a promising strategy, with one of its main uses being the development of nanocarriers for cancer diagnosis and therapy.^1^ Conventional chemotherapeutics are systemically dispersed, have the potential to have serious adverse effects, and can damage both cancerous and healthy cells. Conversely, the design of nanocarriers enables therapeutic delivery and specific targeting of drugs to tumors while preventing drug accumulation in healthy tissues.^2,3^ Most tumor-targeted nanosized carriers depend on passive targeting, which can be accomplished by taking advantage of a solid tumor’s pathophysiological features. The enhanced permeability and retention (EPR) effect within the tumor is a phenomenon caused by the interaction of leaky vasculature and inadequate lymphatic drainage.^4^ Because of the movement of interstitial fluid and the absence of functional lymphatic arteries, this enables nanocarriers between 20 and 200 nm to enter the extravascular region and undergo longer retention durations in the tumor microenvironment.^5−7^

Radionuclides are employed as signal sources in the development of drugs because they can be incorporated into formulations without changing their physical and biological properties. The primary advantage of using labeled formulations in pharmaceutical drug development is that they are highly sensitive and detectable in small amounts.^8,9^ Radiolabeling of nanocarriers is required to track a delivery system using nuclear imaging and evaluate its accumulation *in vivo*.^10−12^ Technetium-99m ([^99m^Tc]Tc), a radioisotope, is a helpful tool for noninvasively assessing the biodistribution of nanocarriers. Because of its chemical and physical properties, including its short half-life (*t*_1/2_ = 6.02 h), low γ energy (140 keV), lack of internal radiation, short radiolabeling time (10–30 min), low price, and commercial viability from the Molybdenum-99 ([^99^Mo]Mo)/[^99m^Tc]Tc generator, [^99m^Tc]Tc has been widely utilized for radiopharmaceutical labeling, particularly in the area of diagnostics.^13^ Compared to other frequently used laboratory radioisotopes, [^99m^Tc]Tc’s short half-life reduces the internal radiation risk and permits a higher intake limit.

Niosomes are hydrated nonionic surfactant monomers that self-assemble into spherical bilayer vesicles.^14^ They have received a great deal of interest as effective nanocarriers to deliver anticancer agents, chemicals, vaccines, proteins, and genes because of their excellent drug delivery capacity, including controlled release, targeted delivery, fewer adverse effects, enhanced bioavailability, and wide formulation variability.^15^ Although niosomes are chemically similar to phospholipid vesicles (liposomes), they have a number of important advantages over them, including affordability, ease of storage, and increased stability (longer shelf life).^16,17^

Several radiochemical processes (direct, chelator-mediated, and encapsulation) can be used to radioactively label nanoparticulate drug delivery systems with a specific radionuclide. One approach is the encapsulation of the radionuclide on the nanocarrier during synthesis. Another is direct incorporation of the radionuclide on the surface or on a chelator that is already attached to the surface.^18^ For labeling drug delivery systems with [^99m^Tc]Tc, the direct labeling method or the chelate labeling method can be used. Direct labeling involves [^99m^Tc]Tc directly forming bonds with the functional groups of the nanocarrier, whereas surface chelation involves the chelation of [^99m^Tc]Tc to a lipid–chelator conjugate incorporated in the bilayer. In a study by De Silva et al.,^14^ niosome formulations were radiolabeled with [^99m^Tc]Tc using diethylene triamine pentaacetic acid (DTPA) as a chelator. According to this study, niosomes were radiolabeled with high labeling efficiency (>90%).^14^ In another study by Almasi et al.,^19^ niosome formulations were radiolabeled with [^99m^Tc]Tc using hexamethyl propylene amine oxime (HMPAO) as a chelator, and the labeling efficiency of [^99m^Tc]Tc-niosomes was found to be >90%. According to the results of an *in vivo* biodistribution study in breast tumor-bearing mice, the [^99m^Tc]Tc-niosome concentration in tumors was found to be significantly higher than [^99m^Tc]Tc-HMPAO at all time intervals. The authors explained the greater affinity of niosomes for cancerous tissue by the extravasation of nanoniosomes into tumor tissue as a result of abnormal and disordered angiogenesis of tumor tissue and the effect of EPR.^19^ In this study, we used the direct labeling method to radiolabel niosome formulations.

The purpose of this research is to investigate the applicability of radiolabeled blank niosomes as a nanocarrier for cancer diagnostic agents. To do this, using the film hydration method, niosome formulations were developed and characterized according to particle size, size distribution (polydispersity index (PdI)), ζ-potential, and image profile. Then, niosomes were radiolabeled with [^99m^Tc]Tc using the ascending radioactive thin-layer chromatography (RTLC) method, and radiolabeled niosomes were subjected to quality control using RTLC and radioactive ultrahigh-performance liquid chromatography (R-UPLC) methods. The stability of radiolabeled niosomes in different mediums and partition coefficient studies were investigated. Finally, in HT-29 cell lines (human colorectal adenocarcinoma), a comparative *in vitro* cell culture experiment of radiolabeled niosomes and reduced/hydrolyzed (R/H)-[^99m^Tc]NaTcO_4_ was performed for the potential use of radiolabeled niosomes in nuclear imaging.

## 2 Materials and Methods

### 2.1 Materials

Sigma-Aldrich provided Span 60, Tween 60, cholesterol, and stannous chloride dihydrate. The [^99^Mo]Mo/[^99m^Tc]Tc generator (Ege University, Türkiye) was used to extract [^99m^Tc]Tc. All chemicals and solvents were provided by Merck (Germany), were of high-performance liquid chromatography (HPLC) or analytical grade, and no additional purification was performed. The HT-29 cell line (human colorectal carcinoma) was obtained from ATCC. Gibco Invitrogen (Grand Island, NY) purchased all cell culture reagents and materials.

### 2.2 Preparation of Niosomes

The niosome formulations were prepared *via* the film hydration method.^20,21^ Briefly, cholesterol (38.66 mg) and surfactants [Span 60 (26.6 mg) and Tween 60 (50.16 mg)] were weighed and dissolved in chloroform (10 mL) in a round-bottom flask. The chloroform was allowed to evaporate in a rotary evaporator (IKA RV 8V, Türkiye) at 100 rpm at 60 °C. The resulting films were hydrated with pH 7.4 phosphate buffer at 100 rpm at 60 °C for 1 h, and particle size reduction was performed by sonication (Qsonica LLC, Newton, CT) at 350 W, 30 s cycle, for 15 min. The formed niosomes were precipitated by ultracentrifugation (Optima XE-90, Beckman Coulter, Brea, CA) at 20,000 rpm for 1 h, and this process was repeated three times. Finally, for lyophilization, 3% trehalose solution was added to the washed niosome formulation, and the system was frozen at −80 °C and lyophilized using a LaboGene Scanvac CoolSafe freeze dryer device (Scandinavia) for 48 h.

### 2.3 Characterization of Niosomes

#### 2.3.1 Particle Size, PdI, and ζ-Potential of Niosomes

The mean particle size and distribution of niosome formulations were determined with dynamic light scattering (DLS) using the Malvern Zetasizer Nano ZS (Malvern Instruments, Malvern, U.K.). The DLS analysis was performed at a detector angle of 173° at 25 °C. Before analysis, the niosome formulations were diluted with ultrapure water (1:400). The particle size and PdI value measurements were carried out five times, and the results were presented as the mean (nm).

The ζ-potential value of niosome formulations was determined using the Malvern Zetasizer Nano ZS (Malvern Instruments) using a folded capillary cell (DTS1070). Before analysis, the niosome formulations were diluted with ultrapure water (1:400). ζ-Potential measurements were carried out five times, and the results were presented as ζ-potential (mV).

#### 2.3.2 Morphological Features of Niosomes

Scanning electron microscopy (SEM) was used to analyze the niosome formulations’ visual characteristics. To achieve this, dried niosomes were mounted onto an aluminum grid and coated with gold using a vacuum evaporator (K550X Sputter Coater, EMITECH) at a thickness of 10 nm for 1.5 min under conditions of 15 mA and 6 × 10^–2^ mbar. The coated nanoparticles were then scanned using a SEM device (Philips XL-30S FEG) under conditions of 100,000× magnification and 7.5kV.

### 2.4 Stability of Niosomes

At 5 ± 3, 25 ± 5 °C, and 60 ± 5% relative humidity (RH), 40 ± 5 °C and 75 ± 5% RH, the stability of niosomes was examined over a period of 6 months. The formulations’ DLS, ζ-potential, and physical properties were assessed. All values were statistically compared. The stability studies of niosome formulations are ongoing.

### 2.5 Radiolabeling of Niosomes

The niosomes were radiolabeled with [^99m^Tc]Tc using different amounts of stannous chloride (10, 25, 50, 100, 500, and 1000 μg mL^–1^, in distilled water), which was a reducing agent.^10,22^ [^99m^Tc]-pertechnetate solution ([^99m^Tc]NaTcO_4_) was eluted from the [^99^Mo]Mo/[^99m^Tc]Tc generator using 0.9% w/v NaCl (SF) solution. Briefly, 0.1 mL of [^99m^Tc]Tc (37 MBq mL^–1^, Atomlab 100 Dose Calibrator, Biodex Medical Systems) was mixed with stannous chloride solution. To this composition, niosomal suspension (1 mL) was added, vortexed for 1 min, and incubated for 15 min. The labeling efficiencies of niosome formulations were assessed using both RTLC and R-UPLC.

#### 2.5.1 RTLC Method

The radiochemical purity (RP) of radiolabeled niosomes was evaluated by RTLC (Bioscan AR 2000) at different time intervals for 6 h. Instant thin-layer chromatography–silica gel-coated fiber sheets (ITLC-SG) and Whatman No. 3 paper were used as the stationary phases, and SF and pyridine:acetic acid:water (PAW; 3:5:1.5) were used as the mobile phases.^14^ The radiochemical purity (RP%) was calculated using the following equation (eq 1)
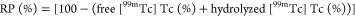
1

#### 2.5.2 R-UPLC Method

The R-UPLC system with a C18 column connected to a photodiode array detector (PDA) and an extra-thallium-doped sodium iodide (NaI(Tl)) γ detector for the [^99m^Tc]Tc compounds was used to evaluate the radiolabeled niosome formulations (wavelength 300 nm). For analytical runs, the flow rate was 1 mL per minute. Chromatographic analysis was performed with a 10 μL reaction mixture. Trifluoroacetic acid (TFA) was used as the eluent in all runs at a concentration of 0.1% in both water and acetonitrile (50:50).^23^

#### 2.5.3 *In Vitro* Stability of Radiolabeled Niosomes

The stability of radiolabeled niosomes was evaluated in SF at 25 °C, serum fetal bovine serum (FBS): phosphate buffer solution (PBS) (pH 7.4) (50:50%, v/v) at 37 °C, and culture medium (McCoy’s 5A) at 37 °C using RTLC. For that, 100 μL of radiolabeled niosome formulations were incubated with 900 μL of SF, serum, and cell medium. The samples were assayed by RTLC to evaluate the stability of radiolabeling.

#### 2.5.4 Partition Coefficient Study of Radiolabeled Niosomes

For the partition coefficient study of [^99m^Tc]Tc-labeled niosomes, *n*-octanol and PBS (pH: 7.4) were used. In a centrifuge eppendorf, n-octanol (500 μL), PBS (450 μL), and radiolabeled formulation (50 μL) were added, mixed for 1 min, then centrifuged at 5000 rpm for 30 min. The mixture underwent centrifugation and was split into two phases. A total of 100 μL of lower and upper phase activity were counted using a γ counter (Sesa Uniscaller). The following equation (eq 2) was used to obtain the log *P* value of formulations
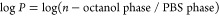
2

### 2.6 Cell Culture Study

HT-29 (ATCC, HTB-3) was grown in McCoy’s 5A supplemented with 10% FBS and 0.5 mg mL^–1^l-glutamine/penicillin in a humidified atmosphere (95%) with 5% CO_2_ at 37 °C. The HT-29 cells were cultured in flasks with a 75 cm^2^ surface area until reaching 85–95% confluence, and they were seeded at a density of 1 × 10^6^ HT-29 cells per well in plates.

#### 2.6.1 Transepithelial Electrical Resistance Measurement

An epithelial voltammeter (EVOM) was used to measure each cell monolayer’s transepithelial electrical resistance (TEER) before and after the experiment (*n* = 6), to determine whether the monolayers were viable. The following equation (eq 3) was used to calculate the TEER value
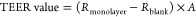
3

where *R*_blank_ is the resistance of the filter membrane, *R*_monolayer_ is the resistance of the cell monolayer along with the filter membrane, and *A* is the membrane’s surface area.^8,24^

#### 2.6.2 Cell Incorporation of Radiolabeled Niosomes

The cell incorporation studies were performed on HT-29 cells, which were human colorectal adenocarcinoma cells, using radiolabeled niosome formulations and R/H-[^99m^Tc]NaTcO_4_ (as a control). For this purpose, niosome formulations and [^99m^Tc]NaTcO_4,_ which contained 18.5 MBq of radioactivity, were incubated with the cells for 30, 60, and 120 min at 37 °C. At the end of the incubation period, first, the culture medium was collected to a centrifuge tube. Then, 0.5 mL of trypsin-EDTA was added to HT-29 cells to collect them. The six-well plates were consecutively washed with 0.5 mL of McCoy’s 5A and 0.5 mL of PBS (pH 7.4) to remove loosely bound surface [^99m^Tc]Tc radioactivity and the cells. The cells were centrifuged at 3000 rpm for 5 min. After that, the cells were placed in another tube while the supernatant was added to the first tube. The radioactivity in cells and supernatant was measured using a γ counter. The percentage of cell incorporation of radiolabeled formulations was calculated by the following formula (eq 4)
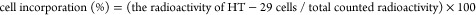
4

### 2.7 Biological Tests of Niosomes

Each parenterally injected radiopharmaceutical must be sterile and pyrogen-free. Biological tests are performed during and after production to ensure that radiopharmaceuticals are sterile and free of pyrogens. For the product to be sterile and free of pyrogens, it must be manufactured under sterile conditions, with sterile environments and materials.

#### 2.7.1 Sterility Test

Sterilization of radiopharmaceuticals can be done by 0.22 μm membrane filtration sterilization under aseptic conditions. In our study, sterile filtration of the final formulation was performed in a sterile cabinet using a 0.22 μm filter.

The British Pharmacopoeia’s direct inoculation method was used to assess the sterility of the niosome formulations. Niosomes were aseptically added to sterilized vials of terrific broth medium (TB medium) and tryptic soy broth medium (TSB medium) before being cultured for 7 days at 37 °C. At the end of the incubation time, the growth of the bacteria in the vials was evaluated.

#### 2.7.2 Pyrogenicity Test

Pyrogens are metabolic wastes of living organisms or nonliving organisms. Pyrogens are typical bacterial endotoxins. They cannot be destroyed in the autoclave and cannot be separated by membrane filtration. Although the solution is sterile, it may contain pyrogen. The way to prevent it is to use high-quality water and chemicals. In our study, we used high-quality water and chemicals and worked in a laminar air flow cabinet.

The pyrogenicity of the niosome formulations was tested using the gel-clot technique in the bacterial endotoxins test (BET). The prepared niosomes and standard endotoxin solution were analyzed comparatively in terms of pyrogenicity. This test is based on the reaction between bacterial endotoxin and the specific lysate. In the presence of endotoxin, the gel is formed *via* a clotting reaction, and the sample failed. The endotoxin limit value of the kit and the maximum valid dilution were estimated according to the European Pharmacopeia 6.0.

#### 2.7.3 Isotonicitiy Test

The final form of the radiopharmaceutical must be isotonic; in other words, the ionic strength must be the same as the blood. Isotonic fluid has the same osmotic pressure as human serum. Radiopharmaceuticals with 250–350 mOsm kg^–1^ are considered isotonic.

An osmometer was used to determine the isotonicity of the niosome formulation. The samples in the eppendorf tube were examined using calibrated equipment.

### 2.8 Statistical Analysis

Results were calculated using the Microsoft Excel program. All experiments were performed at least three times, and the standard deviation (SD) was used to represent the differences within the same group; *p* values less than 0.05 were regarded as statistically significant when using the Student’s *t* test to compare the experimental groups statistically.

## 3 Results and Discussion

### 3.1 Preparation, Characterization, and Stability of Niosome Formulations

Liposomal nanocarriers can effectively encapsulate anticancer agents,^25^ radiopharmaceuticals,^8^ aptamers,^26^ and monoclonal antibodies.^27^ These capsulated agents improve pharmacokinetic stability, exhibit better biodistribution, reduce tissue toxicity, and are delivered to parts of the body not typically accessible to the encapsulated drugs; these properties increase the therapeutic efficacy of a given pharmaceutical.^28^ Among liposomal carriers, niosomes have gained increased attention, and radiolabeled niosomes can be employed as diagnostic agents for conditions like cancer, infection, inflammation, and others. Also, radiolabeled niosomes can be used to examine the pharmacokinetics and biodistribution of niosomes.^19,29^

In this study, niosome formulations were successfully developed and lyophilized using the film hydration method.^20,21^ According to the obtained DLS results, the niosomes were produced with a particle size of 130.5 ± 1.364 nm, and a PdI value of 0.250 ± 0.023. The development method of niosome formulations was simple and incredibly repeatable.

The physicochemical properties of niosome formulations are significant in identifying their *in vivo* performance. A well-designed niosomal system should have a limited PdI value in the nm range for i.v. injection. It has been stated that nanocarriers must have a mean diameter of <200 nm to guarantee the stability of an injectable colloidal formulation.^30^ The degree of uniformity in a particle size distribution is indicated by the PdI value. The PdI scale ranges from 0.0 (totally monodisperse) to 1.0 (highly polydisperse).^31^ A PdI value of 0.3 or less is often regarded as appropriate in the field of drug delivery applications for vesicular systems and represents a homogeneous dispersion of nanoparticles.^32−34^ In this study, the sonication method was used to decrease the particle size and PdI value of niosomes.^35^ So, our results suggest that newly developed niosomal dispersions were quite uniform.

The ζ-potential, which represents the net electrostatic charge on the particle surface, is a crucial factor in determining whether colloidal systems are stable.^36^ The ζ-potential value also influences the interaction of the formulation with the biological system. Compared to uncharged particles, ζ-potentials of less than −30 mV or more than +30 mV can reduce particle aggregation.^37^ In this study, the ζ-potential values of niosomes were −35.4 ± 1.06 mV, which were negatively charged.

**Figure 1:**
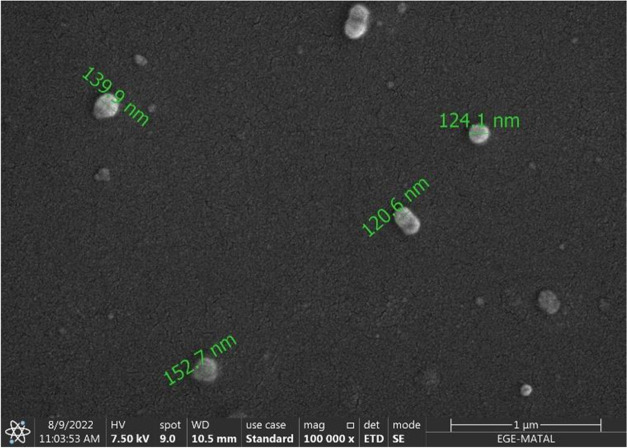
SEM image of niosome formulation. *A Philips XL-30S FEG brand scanning electron microscope device was used in taking this image under 100,000× magnification and 7.50 kV conditions.

The SEM image of niosome formulations is given in Figure 1.

In the obtained image, niosomes were spherical and had a smooth surface. The dimensions of niosomes obtained ranged from 120.6 to 152.7 nm, and the results were compatible with DLS measurement.

**Figure 2:**
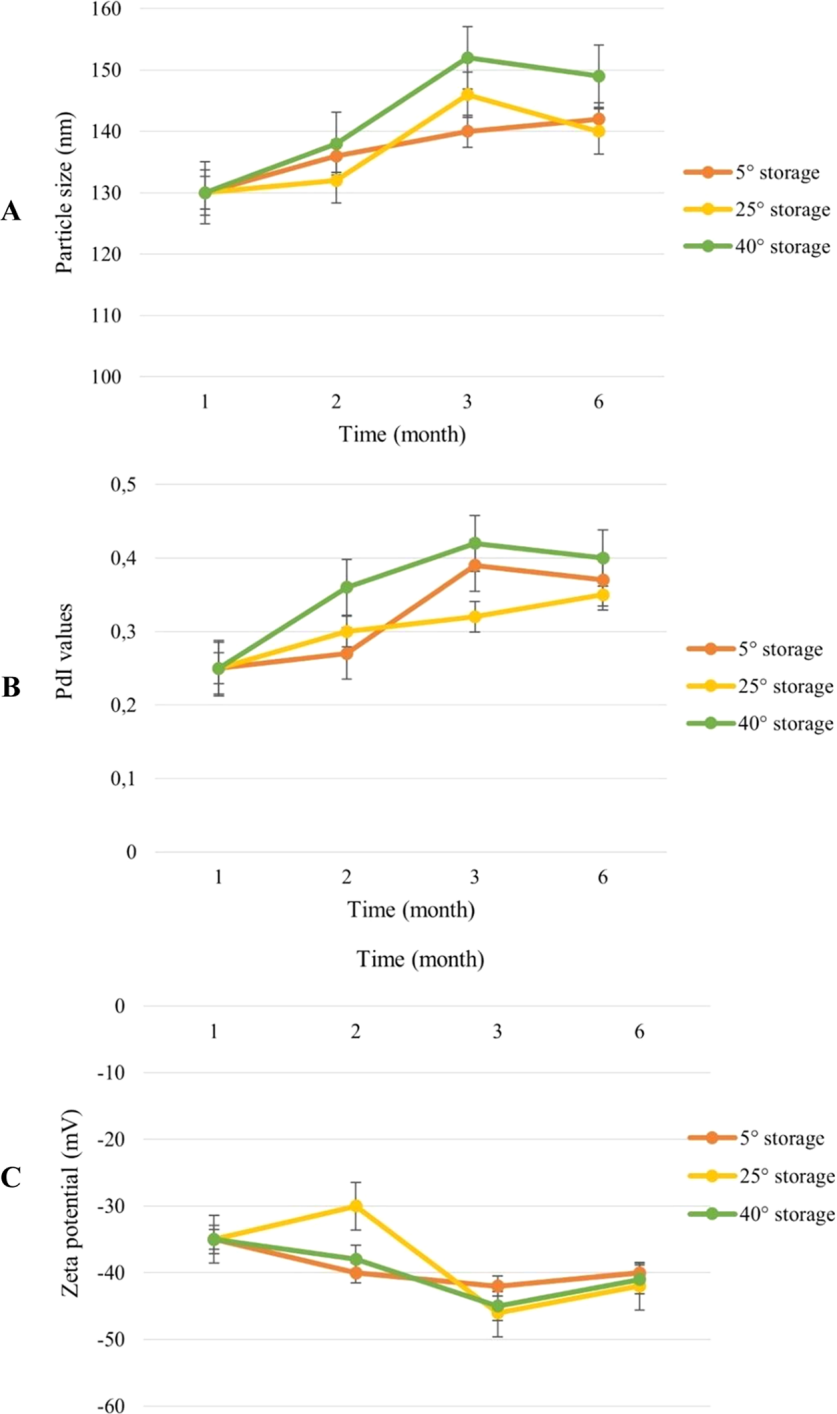
Stability results of niosome formulations (A) particle size, (B) PdI values, and (C) ζ-potential of niosome formulations up to 6 months.

The stability study was performed for niosome formulations at 5 ± 3, 25 ± 5 °C and 60 ± 5% RH, 40 ± 5 °C and 75 ± 5% RH over 6 months, and results are shown in Figure 2. According to the stability results, under all three different conditions, niosome formulations were stable and did not exhibit a substantial change in their particle size, dispersion, or ζ-potential (*p* > 0.05).

### 3.2 Radiolabeling of Niosomes

In this study, the radiolabeling of the niosome formulation was performed using the direct radiolabeling method with [^99m^Tc]Tc radionuclide, which was reduced to lower oxidation states with the reducing agent.^38^

In previous studies, niosome formulations have been labeled with [^99m^Tc]Tc using different chelate agents.^14,19^ Since chelators such as DTPA and HMPAO bind more strongly with [^99m^Tc]Tc, the researchers preferred these methods because the surface chelation method is expected to exhibit higher stability *in vitro* than the direct labeling method. But contrary to expectations, in this study, we achieved high radiolabeling efficiency with the direct labeling method.

The influence of varying amounts of stannous chloride on RP of niosome formulations was shown in Table 1. The amount of stannous chloride added for the reduction of [^99m^Tc]Tc from +7 valencies than +5 valencies was varied from 10 to 1000 μg mL^–1^, while the pH value of the system was constant (pH 7.4). The radiolabeling efficiency of niosome formulation was below 80% when 10 μg mL^–1^ of stannous chloride was added, and it increased significantly to >95% when 500 μg mL^–1^ of stannous chloride was added (*p* < 0.05), different from 25, 50, and 100 μg mL^–1^ of stannous chloride. Further increases in stannous chloride concentration (1000 μg mL^–1^) did not change the percentage of radiolabeling efficiency (*p* > 0.05).

**Table 1:** Radiolabeling Efficiency (%) and Stability of Niosome Formulations Including Different Amounts of Reducing Agent up to 6 h

	stannous chloride amount (μg mL^–1^)					
time (h)	10 μg mL^–1^	25 μg mL^–1^	50 μg mL^–1^	100 μg mL^–1^	500 μg mL^–1^	1000 μg mL^–1^
0.25	79.64 ± 4.91	90.68 ± 2.01	87.12 ± 3.35	93.50 ± 4.23	97.46 ± 2.30	97.05 ± 1.34
1	78.84 ± 4.03	86.52 ± 3.51	73.68 ± 4.21	89.77 ± 3.04	96.74 ± 2.34	97.16 ± 1.03
2	76.54 ± 5.36	74.36 ± 4.31	73.69 ± 4.39	88.77 ± 5.31	96.49 ± 1.98	98.34 ± 2.06
3	73.75 ± 8.12	67.41 ± 6.64	71.75 ± 5.06	84.67 ± 4.58	96.30 ± 1.87	98.20 ± 2.13
4	73.56 ± 6.15	65.17 ± 5.61	69.32 ± 7.34	82.92 ± 5.62	96.06 ± 2.33	97.80 ± 1.68
5	70.55 ± 5.64	62.26 ± 4.38	65.84± 6.31	81.06 ± 6.28	95.88 ± 1.94	97.09 ± 1.42
6	69.96 ± 7.36	62.40 ± 3.58	67.64 ± 5.64	83.49 ± 4.56	95.83 ± 2.36	97.70 ± 1.59

[^99m^Tc]Tc is a mostly used radionuclide to radiolabel nano drug delivery systems. The reducing agent (type and concentration) is the most critical parameter for radiolabeling with [^99m^Tc]Tc. Using high amounts of reducing agent, the colloids occur in the radiolabeling area, and the radiolabeling efficiency decreases. On the other hand, using lower amounts of reducing agent, free [^99m^Tc]Tc is found in the radiolabeling area. In both cases, the radiolabeling efficiency of the system is significantly affected. In radiolabeling studies, mostly stannous salts (chloride, tartrate) are used as reducing agents.^38^ In this study, stannous chloride was used as a reducing agent for niosome formulations. The influence of varying reducing agent amounts was evaluated, and the ideal stannous chloride concentration was found to be 500 μg mL^–1^. When 10 to 100 μg mL^–1^ stannous chloride amounts were used, unacceptable results (radiochemical purity <90%) were obtained. Also, the subsequent addition of an increasing concentration of reducing agent did not have a significant effect on the radiochemical purity of the system (*p* > 0.05). The reason for using 500 μg mL^–1^ stannous chloride is based on the basic principles of general radiopharmacy. So, the least amount of excipient (stoichiometry) was used to ensure sufficient stability. The niosome formulations were incubated with 37 MBq of [^99m^Tc]Tc for 6 h. The loaded amount of [^99m^Tc]Tc in niosome formulations with 500 μg mL^–1^ stannous chloride concentration was found as 36.06 ± 0.01 MBq. Our results showed that 97% of [^99m^Tc]Tc added to the niosomes were loaded into the niosomal formulation.^39,40^

**Figure 3:**
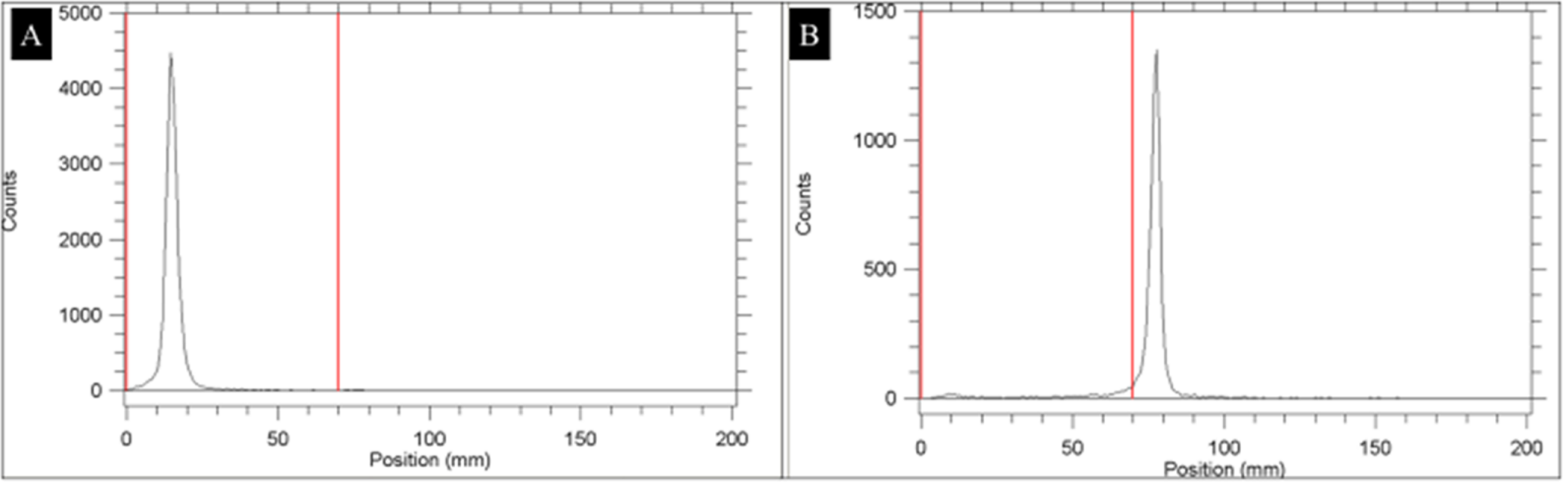
RTLC chromatogram of [^99m^Tc]Tc-niosomes in different mobile phases: (A) SF, (B) PAW; 3:5:1.5. *SF: 0.9% sodium chloride solution, PAW: pyridine/acetic acid/water.

R-UPLC, RTLC, and/or gas chromatography can be utilized for the quality control of radiopharmaceuticals.^41^ To examine the labeling efficiency of [^99m^Tc]Tc-niosomes, first, RTLC approach was employed in this study because it is quick and safe. During the [^99m^Tc]Tc radiolabeling process, three products were formed: [^99m^Tc]Tc-niosomes, [^99m^Tc]NaTcO_4_, and radiocolloids. SF was used as the mobile phase to determine the percentage of [^99m^Tc]NaTcO_4_, which migrated with the solvent front (*R*_f_ = 1.0), while [^99m^Tc]Tc-niosomes and radiocolloids remained at the origin (*R*_f_ = 0.0). A separate developing solvent containing PAW solution (3:5:1.5) was used to identify the percentage of radiocolloids, where radiocolloids stayed at the origin (*R*_f_ = 0.0) while [^99m^Tc]NaTcO_4_ and [^99m^Tc]Tc-niosomes migrated to the solvent front (*R*_f_ = 1.0). Using these systems, the RTLC chromatogram of [^99m^Tc]Tc-niosomes is presented in Figure 3. Under optimized conditions, the RP of [^99m^Tc]Tc-niosomes was over 95% (*p* < 0.05).

**Figure 4:**
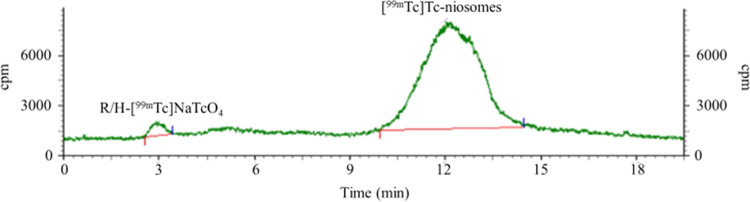
R-UPLC chromatogram of [^99m^Tc]Tc-niosomes.

Second, for the labeling efficiency of [^99m^Tc]Tc-niosomes, R-UPLC was used. The R-UPLC chromatogram is presented in Figure 4. The first peak corresponded to R/H-[^99m^Tc]NaTcO_4_, while the second peak was for [^99m^Tc]Tc-labeled niosomes. The RP of [^99m^Tc]Tc-niosomes was above 95%, acquired *via* RTLC and also R-UPLC.

**Figure 5:**
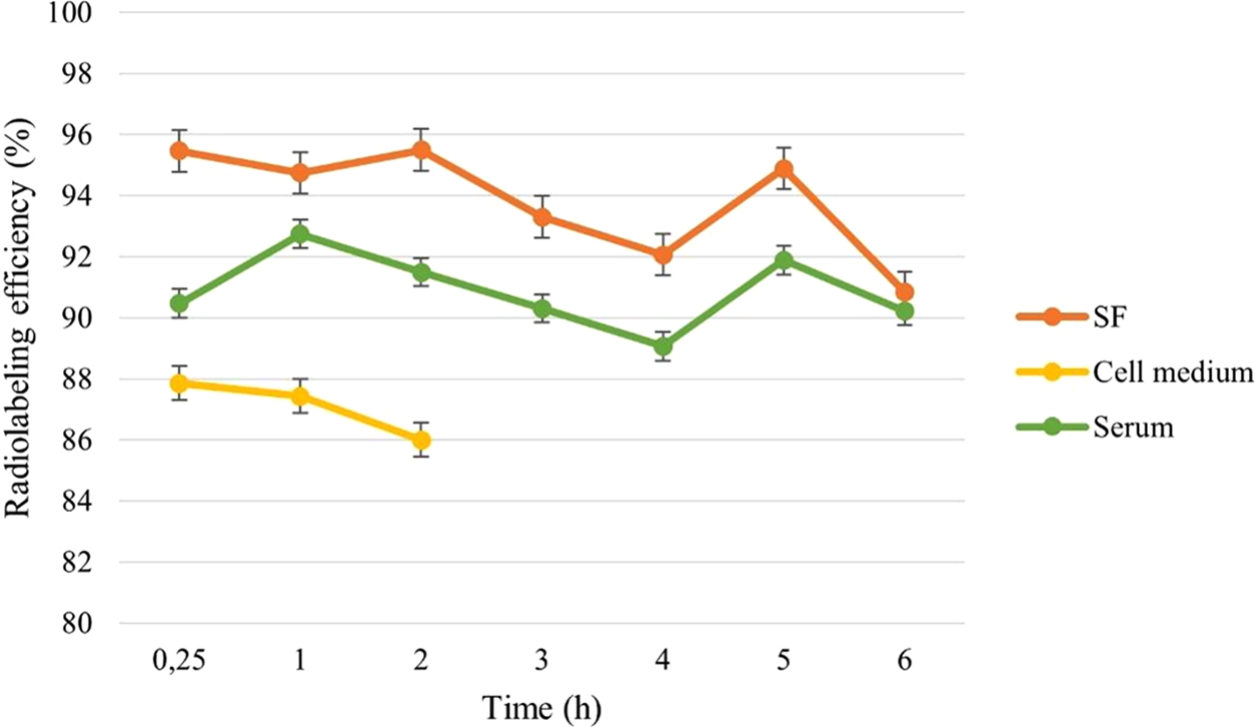
*In vitro* stability of [^99m^Tc]Tc-niosomes in SF, cell medium, and serum. *SF: 0.9% sodium chloride solution, cell medium: McCoy’s 5A supplemented with 10% fetal bovine serum, serum**:** fetal bovine serum: Phosphate buffer solution (pH 7.4) (50/50%, v/v).

The stability of [^99m^Tc]Tc-labeled niosomes was assessed in SF at 25 °C, serum (FBS:PBS (pH 7.4); 50:50%, v/v) at 37 °C, and cell medium at 37 °C (Figure 5). These conditions were chosen to shed light on the application of [^99m^Tc]Tc-niosome formulations in an *in vivo* internal environment at pH 7.4 (physiological pH) and *in vitro* storage.^42^ As [^99m^Tc]NaTcO_4_ is eluted from [^99^Mo]Mo/[^99m^Tc]Tc generator using SF, it is crucial for [^99m^Tc]Tc-niosomes to remain stable in SF. The [^99m^Tc]Tc-niosome formulations were found to be extremely stable in SF, with a high labeling efficiency (>90%) and only a 4.63% decrease in RP after 6 h (Figure 5). This result was comparable to that of Arulsudar et al.,^39^ who developed [^99m^Tc]Tc-liposome formulations by direct labeling for *in vivo* experiments in tumor-bearing mice.^39^

It is essential for [^99m^Tc]Tc-niosomes to maintain their stability throughout the duration of the study to provide an appropriate interpretation of the biodistribution and imaging data of niosomes when administered *in vivo* as a tumor imaging agent.^43^ With reference to this, the [^99m^Tc]Tc-niosomes were also found to be stable in serum solution with a high labeling efficiency (>90%) and did not change the percentage of radiolabeling efficiency during 6 h (*p* < 0.05). This result was found to be quite promising compared to the [^99m^Tc]Tc direct labeling method, as in our method, according to chelate (DTPA) labeling of niosomes.^14^

In addition, radiolabeled niosomes were incubated with cell medium for 2 h. The RP of [^99m^Tc]Tc-niosomes in cell medium was found to be quite stable with >85% RP (*p* < 0.05) (Figure 5). So, our radiolabeled niosome formulation was found suitable for cell incorporation studies.

The log *P* value is considered an indicator of the lipophilicity of a compound or formulation and is calculated in drug development studies to shed light on the drug’s behavior *in vivo*.^44^ A γ counter was used to detect the log *P* of the radiolabeled niosomes and R/H-[^99m^Tc]NaTcO_4_ in this work. The log *P* value of [^99m^Tc]Tc-niosomes was found to be −0.66 ± 0.02 which indicates that [^99m^Tc]Tc-niosomes were slightly hydrophilic in nature (log *P* < 1) and results were found to be consistent with previous studies.^22^ Also, the log *P* value of R/H-[^99m^Tc]NaTcO_4_ (calculated as a control) was found to be −2.131 ± 0.094 which is also known to have very polar properties.

### 3.3 Cell Culture Study of Radiolabeled Niosomes

TEER was used to measure the cells’ resistance both before and after the experiments. This technique, which is frequently employed as a general criterion of cytotoxicity, addresses various functional features of cell structure.^45,46^ In this study, HT-29 cells were used for examining the incorporation affinity of [^99m^Tc]Tc-niosomes. At the start of the experiments for the HT-29 cell line, the TEER values were determined to be between 1197 ± 34.37 and 1272 ± 60.48 Ω cm^–1^. The TEER values after each test period were found to be in the range of 1215 ± 30.48 and 1278 ± 61.60 Ω cm^–1^ (Table 2). During the experiments, there was no significant change in the TEER measurements. The TEER variances did not exceed 40%. The cells were not damaged if the TEER variance never reached 40%.^8,45^ This situation showed that the HT-29 cells were still alive after all studies were finished.

**Table 2:** TEER Values of HT-29 Cell Line during the Experimental Period (*n* = 6) (*p* < 0.05)

time (min)	TEER value (Ω cm^–1^)
0	1215 ± 30.48
30	1246 ± 42.34
60	1278 ± 61.60
120	1267 ± 55.36

**Figure 6:**
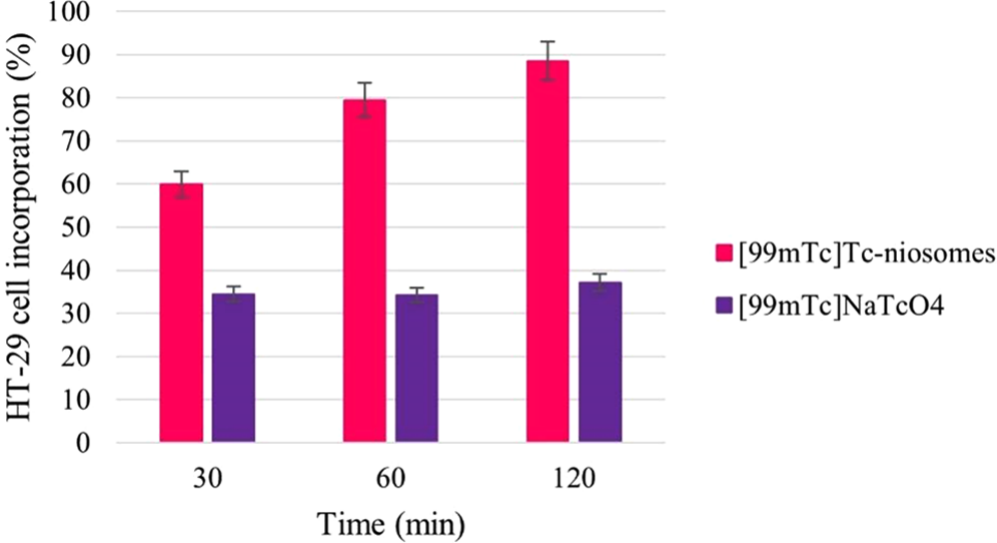
Ability of [^99m^Tc]Tc-niosomes and R/H-[^99m^Tc]NaTcO_4_ to incorporate into the HT-29 cell line.

In recent years, *in vitro* cell culture studies have become increasingly relevant in evaluating the cancer-binding affinities of radioactive compounds or formulations to shed light on *in vivo* studies.^10,22,47^ In this study, the capacity of radiolabeled niosomes to bind to HT-29 cells was investigated. The tests were evaluated for 2 h due to the available half-life of [^99m^Tc]Tc. The cell incorporation percentages to HT-29 cell lines of [^99m^Tc]Tc-niosomes and R/H-[^99m^Tc]NaTcO_4_ (as a control group) are shown in Figure 6.

In addition to avoiding radiation damage to nontarget tissue, the high binding ratio of radiolabeled molecules and systems in the target tissue enables us to collect high-quality images. A low target/nontarget ratio can localize in nontargeting organs and harm these tissues while also affecting the quality of target organ images.^48^ As seen in Figure 6, [^99m^Tc]Tc-niosomes had greater cell incorporation activity on HT-29 cells than R/H-[^99m^Tc]NaTcO_4_ during experimental time *via* passive targeting. The cell incorporation percentage of [^99m^Tc]Tc-niosomes ranged from 59.92 ± 2.43% at 30 min to 88.45 ± 3.54% at 120 min.

Also, to control the study, the cell incorporation percentage of R/H-[^99m^Tc]NaTcO_4_ ranged from 34.43 ± 1.56% at 30 min to 37.14 ± 1.78% at 120 min. This finding demonstrates that our radiolabeled niosomes reacted differently in cell medium than R/H-[^99m^Tc]NaTcO_4_ and verified the high labeling efficiency and *in vitro* stability. Also, in cell incorporation studies in cancer cells using [^99m^Tc]NaTcO_4_ as a control group, it was observed that the uptake of [^99m^Tc]NaTcO_4_ was found to be approx. 60% in MCF-7 (breast cancer cell line) cells, 50% in MDA-MB-231 (triple negative breast cancer cell line) cells at 120 min.^49^ Although it was observed that [^99m^Tc]NaTcO_4_ also showed uptake in cancer cells, in our study, it was observed that the radiolabeled niosome formulation showed much higher uptake than the control group.

Passive targeting relies on the pathophysiology of diseases such as cancer and the EPR property of damaged tissue. Various differentiations occur around the tumor in the regions of cancer tissue, and the most important of these is the EPR effect. When drug molecules are applied to the body without being loaded into any dosage form, they distribute through passive diffusion throughout the body. This situation has now been resolved thanks to the development of nanoparticle drug delivery systems. While nanoparticular systems cannot pass from tightly arranged intercellular spaces in healthy vascular tissue to intercellular fluid, they can easily go out of the vessel in the tumor region where the EPR effect has occurred and whose intercellular spaces reach 500–600 nm dimensions. In this way, only the transition of the drug into the intercellular fluid in the tumor tissue is ensured.^10,22^ So, the mechanism of cell uptake of [^99m^Tc]Tc-niosomes can be explained by the EPR effect. Also, the cell incorporation of [^99m^Tc]Tc-niosomes can be explained by the lipophilicity of the niosome formulation and the natural attraction of lipids by the cells, so niosomes have more penetration through the cytoplasmic membrane than R/H-[^99m^Tc]NaTcO_4._

The therapeutic agents can be added to the niosomes for a variety of uses. To fully understand the biodistribution and pharmacokinetics of such a system, detailed characterization and research should be done. It should be highlighted that some features of the niosomes may change after they are loaded with therapeutic agents. Although the current study has limited data on blank niosomes, according to research by Fu et al.,^50^ tocotrienol-loaded niosomes did not significantly differ from empty niosomes in terms of particle size and ζ-potential.^50^ Since a nanocarrier’s physicochemical characteristics, such as size and charge, have a significant impact on its biodistribution and pharmacokinetic profile, it is anticipated that drug-loaded niosomes will behave similarly to the constructed blank niosomes.^14^

### 3.4 Biological Tests of Niosomes

#### 3.4.1 Sterility Test

The sterility of the niosome formulations was tested. The results of the sterility test showed that the vials were sterile because there was no observable development of bacteria within.

#### 3.4.2 Pyrogenicity Test

According to the pyrogenicity test, niosome formulations were found to be apyrogenic.

#### 3.4.3 Isotonicity Test

The isotonizing of the niosome formulations was determined by the British Pharmacopeia to be 303 mOsm mL^–1^, which was appropriate for injectable formulations.

## 4 Conclusions

In conclusion, [^99m^Tc]Tc-niosomes have been successfully developed as potential nanocarriers for nuclear medicine imaging. The radiolabeling study suggested that 500 μg mL^–1^ stannous chloride was the ideal amount of reducing agent required to radiolabel niosomes. The RP of [^99m^Tc]Tc-niosomes was measured by RTLC and found to be higher than 95%. The radiolabeled niosomes were found quite stable in different media, such as SF, serum, and cell medium, for up to 6 h. The log *P* value of [^99m^Tc]Tc-niosomes was found to be −0.66 ± 0.02. Compared to R/H-[^99m^Tc]NaTcO_4_ (34.18 ± 1.56%), the incorporation percentages of [^99m^Tc]Tc-niosomes (88.45 ± 2.54%) were shown to be higher in cancer cell lines. So, the newly developed [^99m^Tc]Tc-niosomes showed good prototypes for potential *in vivo* use in nuclear imaging in the future.
